# Direct Measurement of Lateral Force Using Dual Cantilevers

**DOI:** 10.3390/s120303200

**Published:** 2012-03-07

**Authors:** Makoto Ishikawa, Masaya Ichikawa, Kouji Miura

**Affiliations:** Department of Physics, Aichi University of Education, Hirosawa 1, Igaya-cho, Kariya 448-8542, Japan; E-Mails: s2080879@auecc.aichi-edu.ac.jp (M.I.); kmiura@auecc.aichi-edu.ac.jp (K.M.)

**Keywords:** lateral force, dual cantilevers, piconewton

## Abstract

We have constructed an experimental system to measure a piconewton lateral force using dual cantilevers which cross with each other. The resolution of the lateral force is estimated to be 3.3 p ± 0.2 pN, which is comparable to forces due to thermal fluctuation. This experimental apparatus works so easily that it will enable us to determine forces during nano-manipulation and nano-tribological measurements.

## 1. Introduction

Measurements of lateral force using scanning probe microscopy have been of great importance in nanoscience and nanotribology [1], because it is necessary to know force needed to move small objects on a surface and to obtain friction force on an atomic scale [2]. However, it is difficult to obtain a correct lateral force by using the conventional lateral force microscope (LFM) because it generally uses the torsion of a micro-cantilever to measure lateral force [3–6]. Here, we propose a novel method to obtain a lateral force by using dual cantilevers which cross with each other.

## 2. Experimental Section

**Figure 1.**
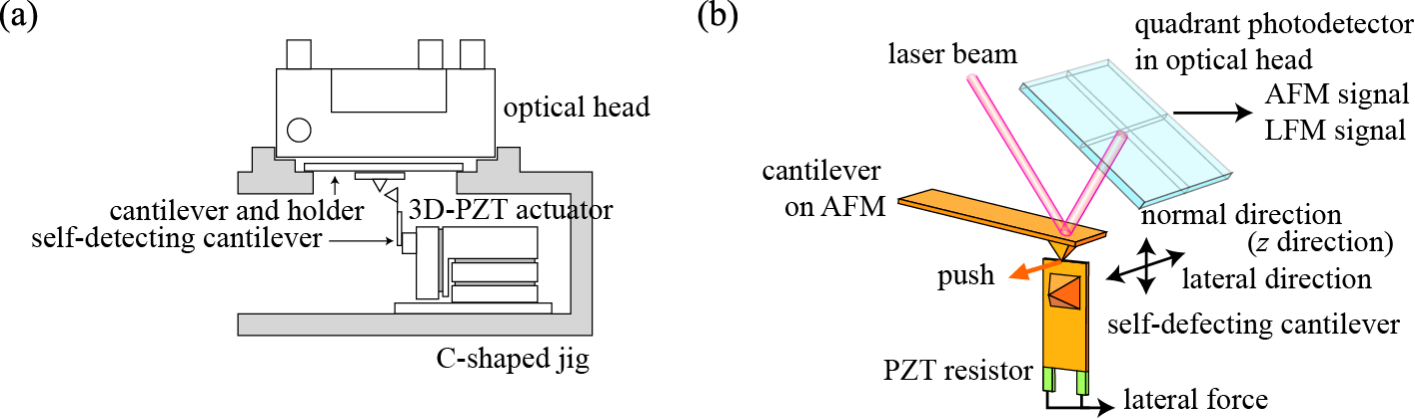
(**a**) Schematic of experimental apparatus. (**b**) Cantilever on AFM and self-detecting cantilever arranged perpendicular to each other.

The overview of experimental setup is depicted in Figure 1(a). This novel setup consists of a 3D-PZT driven actuator (Unisoku Co. Ltd., Japan) and a self-detecting cantilever (NPX1CTP003, SII) as a force sensor on the commercial AFM (SPI-3000, Seiko Instrument, Japan). The self-detecting cantilever attached to the 3D-PZT driven actuator is fixed to the cantilever holder and optical head of the AFM using a C-shaped jig. The 3D-PZT driven actuator has two modes of inertia driven motion and an extension of the 3D-PZT block, which perform the coarse and fine movements for any direction, respectively. The maximum traveling length of the inertia driven motion is 2 mm. The self-detecting cantilever has PZT resistors which act as a small strain gauge. Thus, the force acting on the self-detecting cantilever can be changed into the resistance by supplying the voltage to the bridge circuit, the details of which have been described in a previous paper [7]. The cantilever on the AFM and the self-detecting cantilever are fixed perpendicular to each other so that the bending direction of the self-detecting cantilever is arranged parallel to the torsional direction of the cantilever on AFM, as illustrated in Figure 1(b), where the edge of the self-detecting cantilever comes into contact with the top of the cantilever tip on the AFM. Thus, the bending force acting on the self-detecting cantilever is the same as the lateral force acting on the tip on AFM. The signals of normal and lateral forces acting on the cantilever on the AFM are output from the monitor terminals on the AFM system. On the other hand, another computer controls the movement of the self-detecting cantilever and detects the output signal from the self-detecting cantilever. The experimental apparatus works so easily that its performance is excellent.

## 3. Results

**Figure 2.**
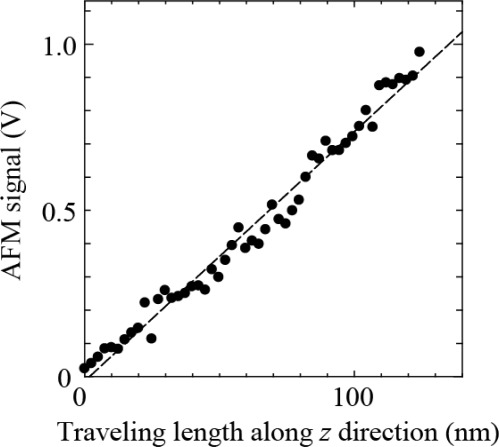
AFM signal *vs.* traveling length along *z* direction.

First, the self-detecting cantilever is moved to the bottom of the cantilever tip on the AFM by using the inertia driven motion of the 3D-PZT driven actuator under the optical microscope to check the experimental setup. The self-detecting cantilever is carefully moved up until the signal of normal force from the cantilever on the AFM changes. After that, the self-detecting cantilever is driven up and down by using the extension of the PZT block to measure the sensitivity along the normal direction (z direction) of the self-detecting cantilever. Figure 2 shows the AFM signal *versus* the traveling length along the z direction of the self-detecting cantilever, which clearly exhibits their proportional relationship. The sensitivity was estimated to be 7.5 mV/nm from the slope of Figure 2, which is consistent with one obtained by using the AFM. Hence it is confirmed that the movement along the z direction of the self-detecting cantilever works well.

**Figure 3.**
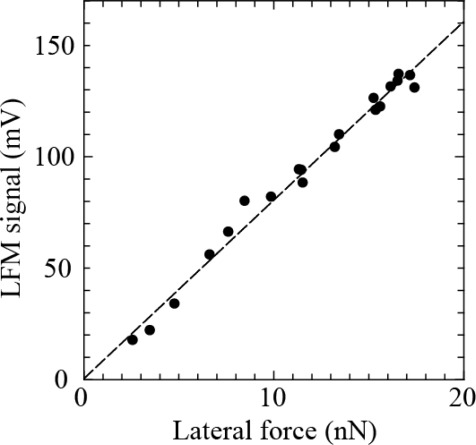
LFM signal *vs.* lateral force.

Next, the self-detecting cantilever is driven along the lateral direction of the cantilever on the AFM while monitoring the output of the LFM signal. Figure 3 shows the LFM signals *versus* lateral force, which clearly exhibits their proportional relationship. The sensitivity was estimated to be 8.0 mV/nN ± 0.6 mV/nN from the slope of Figure 3, which is comparable to that presented by Schwarz *et al*. [8]. Thus, the resolution of the lateral force can be estimated to be 3.3 pN ± 0.2pN because the resolution of the LFM signal is 26.67 μV, which is comparable to forces due to thermal fluctuation. Hence it is found that this method has the potential to measure a lateral force of piconewtons.

## 4. Conclusions

In this paper, we have proposed the novel method to detect a piconewton lateral force using dual cantilevers which cross with each other. This experimental apparatus works so easily that its performance is excellent. This novel method will enable us to determine forces during nano-manipulation and nano-tribological measurements.
